# Self-report of psychological symptoms in hypoparathyroidism patients on conventional therapy

**DOI:** 10.20945/2359-3997000000041

**Published:** 2018-05-07

**Authors:** Amanda J. Arneiro, Bianca C. C. Duarte, Rodrigo M. Kulchetscki, Victor B. S. Cury, Maicon P. Lopes, Breno S. Kliemann, Ileana B. Bini, Marcos Assad, Gleyne L. K. Biagini, Victoria Z. C. Borba, Carolina A. Moreira

**Affiliations:** 1 Universidade Federal do Paraná Universidade Federal do Paraná Serviço de Endocrinologia e Metabologia Divisão de Endocrinologia Curitiba PR Brasil Divisão de Endocrinologia, Universidade Federal do Paraná (UFPR), Serviço de Endocrinologia e Metabologia (SEMPR), Curitiba, PR, Brasil; 2 Hospital Universitário Evangélico de Curitiba Hospital Universitário Evangélico de Curitiba Divisão de Endocrinologia Curitiba PR Brasil Divisão de Endocrinologia, Hospital Universitário Evangélico de Curitiba, Curitiba, PR, Brasil; 3 Universidade Federal do Paraná Universidade Federal do Paraná Departamento de Medicina Interna Curitiba PR Brasil Departamento de Medicina Interna, Universidade Federal do Paraná (UFPR), Curitiba, PR, Brasil; 4 Fundação Pró-Renal Setor de Histomorfometria Óssea Laboratório PRO Curitiba PR Brasil Laboratório PRO, Setor de Histomorfometria Óssea, Fundação Pró-Renal, Curitiba, PR, Brasil

**Keywords:** Hypoparathyroidism, psychological distress, psychopathological symptoms, SCL-90-R, parathyroid hormone

## Abstract

**Objective:**

Hypoparathyroidism is characterized by parathyroid hormone deficiency and hypocalcemia. It has been demonstrated that these patients may also present psychiatric symptoms and decrease of quality of life. The aims of this study were to evaluate the presence of psychopathological symptoms in a cohort of patients with hypoparathyroidism and compare to a control group.

**Subjects and methods:**

Patients were submitted to a cross-sectional Symptom Checklist-90-R (SCL-90-R) questionnaire that evaluates psychopathological symptoms by means of the Global Severity Index (GSI), Positive Symptoms Total (PST) and Positive Symptom Distress Index (PSDI). A score based in the positive symptoms was calculated (T-score). The test group was composed of patients with hypoparathyroidism, and control by thyroidectomized patients without hypoparathyroidism. A correlation between the presence of psychological symptoms and clinical features was analyzed.

**Results:**

The study included 57 patients with a mean age of 51.1 ± 16.4 years; 20 as a control and 37, test group. There were no differences between groups regarding gender, mean age and age at diagnose. Hypoparathyroidism patients presented higher GSI index than the control group (p = 0.038). Mean T-score of the test group was as elevated as 58.2 ± 5.3 (reference range < 55). No correlation of the number of psychological symptoms to clinical and laboratorial parameters was observed.

**Conclusion:**

Patients with hypoparathyroidism attending our outpatient clinics presented an increase in the number of self-report of psychological symptoms when compared with a control group. However, no correlation with hypocalcemia and clinical parameters was observed. Future studies are necessary to evaluated if the absence of PTH play a role on it.

## INTRODUCTION

Parathyroid hormone (PTH) is the main regulator of calcium metabolism. It maintains calcium homeostasis by directly regulating bone remodeling and renal excretion, and indirectly regulating the production of 1,25-dihydroxyvitamin D. Deficiency of PTH leads to hypoparathyroidism, a metabolic disorder in which the diagnosis is confirmed by the occurrence of hypocalcemia (1). Although several etiologies may be associated with hypoparathyroidism, in approximately 75% of the patients the disease occurs after surgery in the anterior cervical region (thyroidectomy and parathyroidectomy) (2).

Hypoparathyroidism is a rare disorder, and only a few studies have directly assessed its prevalence. Estimates point out to approximately 77,000 cases (3) and 37/100,000 person-years (4) in the United States, and 22/100,000 person-years in Denmark (5).

Treatment of hypoparathyroidism includes supplemental calcium at high doses and calcitriol or other vitamin D analogs. The main morbidities affecting these patients are due to the occurrence of hypocalcemia and hyperphosphatemia and the adverse effects of long-term use of calcium and vitamin D treatment such as nephrolithiasis, nephrocalcinosis, and renal function impairment (1). Studies have shown that patients with hypoparathyroidism present a variety of psychological and neurological manifestations (6–9) and a high prevalence of psychiatric morbidities (7,8), including an increased incidence of depression and bipolar disorder (7) and a greater risk of hospitalization due to neuropsychiatric conditions (8). Decreased cognitive function has also been demonstrated in these patients (9). In an online interview with over 300 adults with hypoparathyroidism, more than 50% of the respondents reported cognitive and emotional symptoms (6), including mental lethargy (72%), memory loss (65%), sleep disturbances (57%), anxiety (59%), and depression (53%).

Although evidence shows frequent psychological and neurological manifestations interfering with the quality of life of patients with hypoparathyroidism, these manifestations have not been carefully detailed, and are not usually identified by physicians caring for these patients. In a US study including patients with permanent hypoparathyroidism following thyroidectomy, 47% of the respondents reported feeling that their health was “much worse” after surgery (10). In contrast, only 16% of the surgeons who had operated on these patients estimated that the patients were dissatisfied after surgery. The authors then concluded that the impact of postoperative hypoparathyroidism on a patient's quality of life is actually underestimated (10).

The aims of this study were to evaluate the presence of psychopathological symptoms, which is related to well being and mood, in a cohort of patients with hypoparathyroidism undergoing conventional treatment with calcium and vitamin D, and analyze the association between these symptoms to other clinical factors including disease duration and serum calcium levels.

## SUBJECTS AND METHODS

In this cross-sectional, case-control study, we conducted an active search of medical records of patients attending the Thyroid Cancer and Bone Metabolism outpatient clinics at the Endocrine Division at Federal University of Paraná (SEMPR) to identify patients with hypoparathyroidism and controls without hypoparathyroidism. We identified 57 patients who were further contacted by phone and invited to participate in the study. All patients who agreed to participate signed an informed consent form. The study was approved by our local Ethics Committee.

The study groups consisted of 37 patients with postsurgical or autoimmune hypoparathyroidism and 20 control patients without hypoparathyroidism who had undergone total thyroidectomy. The majority of the patients were underwent to surgery in the last 23 years by the head and neck surgery team of the *Hospital de Clínicas*, Federal University of Parana. Exclusion criteria comprised a duration of hypoparathyroidism shorter than 6 months and a history of psychiatric or neoplastic diseases, except for thyroid neoplasms.

We performed a detailed review of the medical records of the participants to gather information regarding their age, gender, etiology, disease duration, age at diagnosis, and doses of medications in use. In addition, we obtained the patients’ serum levels of calcium, phosphate, 25-hydroxyvitamin D, and PTH, collected on average 6 months after surgery, and calculated their mean levels and standard deviations. To calculate the mean levels of serum calcium, phosphate, and PTH, we analyzed the first and last visits of each patient, as well as three intermediate visits (a total of five time points). We have previously published the mean treatment doses of calcium carbonate, calcitriol, and cholecalciferol used by the patients at five time points during their follow-up (11).

Patients in both groups filled out the Symptom Checklist-90-R (SCL-90-R) questionnaire, an internationally and nationally validated (12) tool. The SCL-90-R was chosen, since it is the most complete questionnaire to evaluated psychopathological symptoms and also has been previously used in well-recognized study in patients with hypoparathyroidism receiving conventional therapy (13). The questionnaire comprises 90 items, each item graded on a scale from 0 (none) to 4 (extreme). The patient must choose the grade (number) that best corresponds to how much they have worried or how much distress they have experienced related to that item over the previous 7 days. After receiving instructions, the patients filled out the questionnaires individually.

The SCL-90-R assesses presence of psychopathological symptoms of an individual by evaluating three global indices of distress: the Global Severity Index (GSI), the Positive Symptom Total (PST), and the Positive Symptom Distress Index (PSDI) (12,14,15). GSI is considered the best indicator to assess the current level of the disorder, and is calculated by adding up the obtained values and dividing the result by the number of items answered, thus reflecting the quality of life in general. The PST assesses the number of symptoms, and is calculated by the number of items scored above zero. The PSDI measures the intensity of symptoms and is calculated by dividing the sum of the items answered by the number of items scored above zero. The latter is also used to assess the patient's response style, or in other words, if they tend to “augment” or “attenuate” their symptoms (12,14,15). Lastly, the SCL-90-R evaluates nine dimensions of psychological symptoms, including somatization, obsessive-compulsive traits, interpersonal sensitivity, depression, anxiety, hostility, phobic anxiety, paranoid ideation, and psychoticism (12,14,15). These nine dimensions of psychological symptoms of the SCL-90-R were not evaluated in the present study.

We converted the GSI responses to T-scores in order to compare these results with those in the literature. The classification is based on T-score values, as follows: T-scores between 40 and 55 are within the normal range, those greater than 55 indicate already significant impairment of mood, while those above 60 are considered pathological, equivalent to severe impairment of well-being and mood (12,14,15).

### Statistical analysis

We analyzed the data with the software R, version 3.2.3 (16). The normality of the data was evaluated with the Shapiro-Wilk test. To evaluate statistical differences between groups of data following a normal distribution (serum calcium), we used the parametric Student's *t* test, and for data without a normal distribution (current age, age at diagnosis, disease duration, etiology, phosphate, vitamin D, and PTH), we used the nonparametric Wilcoxon-Mann-Whitney test. To evaluate whether the proportion of men and women between the groups differed statistically, we used Fisher's exact test. Student's *t* test was also used to analyze the sum of the items of the questionnaire, as well as the scores obtained for each of the indices – GSI, PST, and PSDI. To assess the correlation between quality of life and disease duration, age at diagnosis, etiology, and serum calcium and phosphate, we used Spearman's rank correlation coefficient, because the data followed a non-normal distribution. We considered p values < 0.05 as statistically significant.

## RESULTS

The study included 57 patients with a mean age of 51.1±16.4 years. In total, 20 patients were allocated to the control group and 37 to the hypoparathyroidism group. In the latter, 31 patients had postsurgical hypoparathyroidism, and six had autoimmune hypoparathyroidism. There were no significant differences between groups regarding gender, mean age, mean age at diagnosis, and education level. In general, patients with hypoparathyroidism were asymptomatic except by presenting occasional paresthesia and cramps. Table 1 presents the laboratory parameters in both groups. As expected, the serum levels of calcium, phosphate, and PTH differed significantly between the groups, with patients with hypoparathyroidism presenting lower calcium and higher phosphate levels when compared with those in the control group.

**Table 1:** Characteristics of the study population regarding clinical and laboratory parameters

Parameters	Control	Hypoparathyroidism	P value
Gender^1^	19 women 1 man	34 women 3 men	1
Current age (years)^2^	52.7 ± 14.9	50.2 ± 17.3	0.535
Age at diagnosis (years)^2^	43.0 ± 17.1	38.0 ± 18.6	0.425
Disease duration (years)^2^	8.4 ± 7.3	13.1 ± 5.9	0.025*
Calcium (mg/dL)^3^	9.3 ± 7.3	8.4 ± 1.0	0.003*
Phosphate (mg/dL)^2^	3.6 ± 0.4	8.4 ± 1.0	< 0.001*
Vitamin D (mg/dL)^2^	33.6 ± 11.1	50.2 ± 22.2	0.131
PTH (pg/mL)^2^	49.5 ± 15.7	8.9 ± 4.7	< 0.001*

1 Statistical analysis with Fisher's exact test.

2 Mann-Whitney statistical analysis.

3 Student's *t* test statistical analysis.

* Statistically significant difference.

The values are expressed as mean ± standard deviation.

Patients in the hypoparathyroidism group received conventional replacement therapy with calcium and vitamin D. The mean daily doses prescribed were 1.321 ± 705 mg of calcium carbonate, 0.5 ± 0.28 mcg of calcitriol, and 25500 ± 15000 IU of cholecalciferol.

Table 2 shows the SCL-90-R scores. When compared with control patients, those with hypoparathyroidism had greater GSI scores (1.1 ± 0.5 and 0.8 ± 0.4, respectively, p = 0.03). The significance was maintained when only the post surgical hypoparathyroidism patients (N:31) were compared to the control group (0.8 ± 0.4 vs. 1.1 ± 0.6; p = 0.04). The mean number of the questionnaire items filled out by the patients was higher in the hypoparathyroidism group (93.5 ± 47.3) compared with the control group (84.3 ± 45.2, p = 0.036). No differences were observed between groups in regard to the PST and PSDI subscales (Table 2).

**Table 2:** Indices obtained with the SCL-90-R questionnaire in the controls and hypoparathyroidism groups

SCL-90-R	Control (n = 20)	Hypoparathyroidism (n = 37)	P
Sum of items answered^1^	67.4 ± 36.2	93.5 ± 47.3	0.036
Number of Items Answered^2^	87.7 ± 10.0	87.9 ± 9.4	0.252
GSI (Global Severity Index)^1^	0.8 ± 0.4	1.1 ± 0.5	0.038*
PST (Positive Symptom Total)¹	34.2 ± 16.1	43.3 ± 18.4	0.069
PSDI (Positive Symptom Distress Index)^1^	1.9 ± 0.7	2.1 ± 0.5	0.324

1 Student's t test statistical analysis.

2 Mann-Whitney statistical analysis.

* Statistically significant difference.

The values are expressed as mean±standard deviation.

The mean GSI T-score in patients with hypoparathyroidism was 58.1 ± 5.6. Overall, 27 patients with hypoparathyroidism (72.9%) presented impaired quality of life (T-scores above 55). There was no correlation between GSI scores with age, disease duration, etiology, or levels of serum calcium and phosphate. No correlation between quality of life and disease duration was found.

## DISCUSSION

This study demonstrated that patients with hypoparathyroidism presented status of more psychological distress compared with a control group of individuals without hypoparathyroidism, albeit a correlation with the clinical factors investigated was not confirmed. The significant difference in GSI between the two groups showed that this impairment of mood and in some cases, well-being, might have affected several dimensions of physical and mental symptoms and therefore, quality of life. As demonstrated by the PST scores, patients with hypoparathyroidism showed a tendency to present more symptoms. Regarding the PSDI, the difference between the two groups was also not significant, showing that in spite of the global decrease in well-being, patients with hypoparathyroidism do not tend to amplify the intensity of their symptoms.

A few studies have evaluated of psychological distress and well-being in patients with hypoparathyroidism. Arlt and cols. have published a case-control study with the application of questionnaires assessing a broad-spectrum of well-being parameters in patients with hypoparathyroidism undergoing conventional treatment in Germany (13). In their study, 25 patients with hypoparathyroidism following thyroidectomy were evaluated with the SCL-90-R and other questionnaires. In line with our findings, the authors also observed a significant impairment of mood with a pattern usually seen in anxiety disorders among patients with hypoparathyroidism when compared with thyroidectomized controls without hypoparathyroidism. In both groups, the authors found no correlation between neuropsychiatric assessment scores with serum calcium or disease duration. The mean T-score related to the GSI was only slightly higher than that in our cohort, but it was higher than 55 in both studies, reflecting a significant impairment of mood among patients with hypoparathyroidism across different cultural contexts.

Other studies also assessing patients with hypoparathyroidism undergoing replacement with calcium and vitamin D had different methodologies than ours, including the absence of a control group and lack of a detailed description of treatment type or use of T-scores. These differences hinder a direct comparison of these studies with ours. Despite that, these studies also demonstrated neurological and psychological impairments in this group of patients (6–9).

The causes of the neuropsychiatric changes in patients with hypoparathyroidism are still unclear. Both our study and the one by Arlt and cols. (13) have shown a negative correlation between quality of life assessment scores with serum calcium and disease duration. In addition, most patients undergoing conventional treatment managed to maintain calcium levels within the normal range. Mitchel and cols. periodically measured the serum calcium levels of 120 patients with hypoparathyroidism undergoing calcium and vitamin D replacement for 7 years, and demonstrated that 71% of the patients achieved an acceptable range of calcemia for the disease, considered by the authors as between 7.5 and 9.5 mg/dL (17).

Considering that the high prevalence of neuropsychiatric manifestations coexists with normal serum calcium levels in large part of the patients, this has led to the question of whether the presence of psychological symptoms and therefore, decrease in quality of life, might not be another consequence of chronic hypocalcemia.

Interestingly, it has been described that the PTH receptor PTH2R is more common in the brain than in other tissues (18). One study has used *in situ* hybridization to study the distribution of this receptor in the brain of primates and humans, revealing that its location suggests that it may be related to areas regulating fear, anxiety, reproductive behavior, and nociception. Moreover, the location of the receptor in the brain in primates and humans is very similar (19). These findings suggest that PTH action in the central nervous system may be important in neuromodulating several behaviors related to general well-being.

Some studies have evaluated patients receiving synthetic PTH replacement, a treatment option that became available in the United States in 2015. Two publications (20,21) have shown improvements in quality of life following replacement with this type of hormone. However, these studies have not approached the relation between serum calcium and well-being scores, as done in our study and in that by Arlt and cols. (13).

Vescini and cols. (20) used the 36-item short-form health survey (SF-36) questionnaire to evaluate 42 patients with hypoparathyroidism before and after PTH (1-34) replacement for 6 months. The authors observed a significant improvement in all domains assessed with the questionnaire, including physical and mental symptoms.

The positive effects of the treatment with PTH (1-84) were confirmed in another study that evaluated 69 patients during 5 years, as part of a cohort study of patients with postsurgical and idiopathic hypoparathyroidism assessed before, during, and after discontinuation of hormone therapy, using the SF-36 questionnaire. After 2 months of treatment with PTH (1-84), the patients showed improvements in quality of life indices, a result that persisted in the last evaluation performed 5 years after treatment start. In addition, 27 patients who discontinued treatment due to side effects experienced decreased well-being at the end of that period when compared with those who maintained the therapy. These patients showed significant improvement in T-scores related to social interaction, vitality, mental health, limitations caused by physical health problems, and general health perception (21).

In summary, treatment with PTH replacement was associated with improved quality of life in some studies with a longitudinal patient follow-up model. Clearly, patients with hypoparathyroidism present some degree of impaired quality of life. The neuropsychiatric complications of the disease are not well recognized and are often underestimated. Nevertheless, at the First International Conference on “The Diagnosis, Management, and Treatment of Hypoparathyroidism” in 2015, researchers have emphasized the quality of life as one of the areas requiring additional research within the next 5 years (22). In fact, more evidence is necessary to determine the relation between these patients’ well-being and their biochemical parameters, as well as their response to different treatments.

In conclusion, patients with hypoparathyroidism attending our outpatient clinics presented an increase in the number of self-report of psychological symptoms when compared with a control group, which was similar to results found in other studies. However, no correlation with hypocalcemia and clinical parameters was observed. Clinicians should recognize the presence of this psychological distress since hypoparathyroidism is a long-term disease that often develops early in life.

Future studies are necessary to improve the understanding of the etiology and pathophysiology of the psychiatric changes associated with this disease. In addition, it is important to elucidate if the absence of PTH play a role on it.
